# Third-Generation Sequencing Reveals LncRNA-Regulated HSP Genes in the *Populus x canadensis* Moench Heat Stress Response

**DOI:** 10.3389/fgene.2020.00249

**Published:** 2020-05-07

**Authors:** Jiahong Xu, Meng Fang, Zhihao Li, Maoning Zhang, Xiaoyu Liu, Yuanyuan Peng, Yinglang Wan, Jinhui Chen

**Affiliations:** ^1^Key Laboratory of Genetics and Germplasm Innovation of Tropical Special Forest Trees and Ornamental Plants, Ministry of Education/Engineering Research Center of Rare and Precious Tree Species in Hainan Province, College of Forestry, Hainan University, Haikou, China; ^2^Hainan Key Laboratory for Biology of Tropical Ornamental Plant Germplasm, College of Forestry, Institute of Tropical Agriculture and Forestry, Hainan University, Haikou, China; ^3^Hainan Key Laboratory for Sustainable Utilization of Tropical Bioresources, College of Tropical Crops, Hainan University, Haikou, China; ^4^School of Life Sciences, Lanzhou University, Lanzhou, China

**Keywords:** *Populus x canadensis* Moench, third-generation sequencing, heat stress, lncRNAs, heat shock protein

## Abstract

Long non-coding RNAs (lncRNAs) regulate plant responses to abiotic stresses. However, the short reads produced by second-generation sequencing technology make it difficult to accurately explore full-length transcripts, limiting the study of lncRNAs. In this study, we used third-generation long-read sequencing technology with the PacBio Sequel and Illumina platform to explore the role of lncRNAs in the heat stress response of *Populus x canadensis* Moench trees. We using 382,034,416 short reads to correct 4,297,179 long reads by resulted in 66,657 full-length transcripts, representing 33,840 genes. Then, 753 putative lncRNAs were identified, including 658 sense lncRNAs (87.38%), 41 long intervening/intergenic non-coding RNAs (lincRNAs) (5.44%), 12 antisense lncRNAs (1.59%), and 42 sense intronic lncRNAs (5.58%). Using the criteria | log_2_FC| ≥ 1 and *q*-value < 0.05, 3,493 genes and 78 lncRNAs were differentially expressed under the heat treatment. Furthermore, 923 genes were detected as targets of 43 differently expressed lncRNAs by *cis* regulation. Functional annotation demonstrated that these target genes were related to unfolded protein binding, response to stress, protein folding, and response to stimulus. Lastly, we identified a lncRNA–gene interaction network consisting of four lncRNAs and six genes [*Heat Shock Protein 82* (*HSP82*), *HSP83*, *Disease Resistance Protein 27* (*DRL27*), *DnaJ family protein* (*DNJH*), and two other predicted protein-coding genes], which showed that lncRNAs could regulate HSP family genes in response to heat stress in *Populus*. Therefore, our third-generation sequencing has improved the description of the *P. canadensis* transcriptome. The potential lncRNAs and HSP family genes identified here present a genetic resource to improve our understanding of the heat-adaptation mechanisms of trees.

## Introduction

High temperature is a common abiotic stress experienced by plants. Approximately 23% of land on the Earth has an annual mean air temperature above 40°C (Leone et al., 2003). The frequent high temperatures that have occurred throughout the world in recent years have seriously threatened the growth of plants (Mora et al., 2015). High temperatures alter plant metabolic processes, thus affecting growth and development. Plant adaptations to acclimatize to high temperature include changes in morphology and physiology. For example, in response to heat stress, plants have shown decreased photosynthesis and transpiration (Mathur et al., 2014), and cell membrane stability (Havaux et al., 1996) and increased levels of antioxidants (Xu et al., 2006; Zou et al., 2011), phytohormones (Abdelrahman et al., 2017), compounds used for osmotic adjustment (Wahid et al., 2007), and heat shock proteins (HSPs) (Miller and Mittler, 2006). The changes at the molecular level take place immediately after the onset of heat stress and result in altered gene expression and transcript accumulation, and the synthesis of HSPs to induce stress tolerance (Schulze et al., 2005). HSPs enhance protein stability by helping other proteins to not unfold during heat stress (Wahid and Close, 2007).

HSPs are mainly divided into six classes based on their molecular weights: HSP100, HSP90, HSP70, HSP60, HSP40, and small HSPs (Kotak et al., 2007). In plants, the expression of many HSP genes increases and regulates the synthesis of HSPs after exposure to heat stress (Ada et al., 1987; Kant et al., 2008). For example, previous transcriptome analysis studies reported similar changes in expression of HSP genes in *Lolium perenne* and *Festuca arundinacea* (Hu et al., 2014; Wang K. et al., 2016). *HSP40* and *HSP70* were up-regulated in *Dianthus caryophyllus* under heat stress (Wan et al., 2015). In addition, transgenic creeping bentgrass (*Agrostis stolonifera*) plants expressing a rice (*Oryza sativa*) SUMO ligase showed increased expression of two *HSP* genes and improved resistance to high temperatures compared with non-transgenic plants (Li et al., 2013). These results suggested that HSPs play an important role in response to high temperature stress in plants.

LncRNAs have many key regulatory functions in plants. For example, a cold-inducible intronic lncRNA silences the floral repressor *FLOWERING LOCUS C* (Heo and Sung, 2011). Moreover, lncRNAs have roles in abiotic stress responses. For example, overexpression of the lncRNA npc536 in *Arabidopsis thaliana* enhances plant salt tolerance (Amor et al., 2009). In addition, the targets of rice stress-induced lncRNAs were enriched in genes involved in development and stress tolerance, suggesting that lncRNAs also play important roles in rice growth under stress (Yuan et al., 2018).

Upon heat stress, lncRNAs play an important role. For example, lncRNA HSR1 associates with protein eEF1A in response to heat stress (Shamovsky and Nudler, 2009). Nucleolar sequestration of HSP72 is facilitated by lncRNA in response to heat stress (Kotoglou et al., 2009). In addition, Transcription elongation of HSPs were regulated by lncRNA, and suppression of transcription after heat stress were mediated by lncRNA (Yakovchuk et al., 2009, 2011; Place and Noonan, 2014). Although there is a lot of research on lncRNA, our understanding of the role of lncRNA in heat stress is very limited. Development of research methods and techniques, especially for the identification and characterization of lncRNAs, will improve our understanding of non-coding RNA functions. Many studies have reported on the response of regulatory lncRNA genes to stress using RNA sequencing (RNA-seq), which enables accurate, genome-wide analysis of transcriptomes (Au et al., 2012). However, high-throughput sequencing produces short reads; the assembly of transcripts from these reads can be substantially improved using the long reads produced by single-molecule long-read “third-generation” sequencing technologies. Indeed, many studies have harnessed third-generation sequencing (Treutlein et al., 2014; Abdel-Ghany et al., 2016; Bo et al., 2016) and found that the resulting full-length sequences are more accurate for exploring gene length and lncRNAs (Roberts et al., 2013; Hackl et al., 2014).

Here, we used single-molecule real-time (SMRT) technology combined with high-throughput sequencing to reveal changes in lncRNA abundance in *Populus x canadensis* Moench after treatment at 40°C for 1 h. To the best of our knowledge, this study using third-generation sequencing to characterize the *P. canadensis* transcriptome and serves as a basis for further research on the response to high temperature.

## Materials and Methods

### Plant Materials and Heat Stress Treatment

For the heat stress treatment, we selected uniformly developed seedlings (one month) of *P. canadensis* and transplanted them into individual pots. The seedlings were grown in a greenhouse at Hainan University (Danzhou; 109°29′ 25″ E, 19°30’ 40″ N) and grown for five months, and six uniform individuals were used for the treatment. The plants were named pch1, pch2, and pch3 (for *P. canadensis* heat) for the heat-stress treatment under 40°C for 1 h, and pcq1, pcq2, and pcq3 (for *P. canadensis* control) for the untreated controls. Leaf samples were collected from the six plants for RNA extraction. The collected leaves were immediately frozen in liquid nitrogen and stored in a −80°C ultra-low temperature freezer until they were used for RNA isolation.

#### RNA Isolation and Illumina Sequencing

Total RNA of mature leaves collected from pcq and pch groups was extracted by CTAB method. The quality of the six RNA samples was assessed by NanoDrop 2000 (Thermo Scientific) and Agilent 2100 Bioanalyzer (Agilent Technologies). Only RNA samples with absorption values in the 260/280 ratio from 1.9 to 2.2, and in the 260/230 ratio ≥2.0, and we used only samples that had an RNA integrity number (RIN) >6.8 for follow-up experiments. Polyadenylated mRNA was enriched by oligo (dT) magnetic beads.

For the short-read RNA-seq, fragmentation buffer was added to fragment the mRNA into shorter pieces. We synthesized single-stranded cDNA from the mRNA using random hexamer primers and synthesized double-stranded cDNAs by adding buffer, dNTPs, and DNA polymerase I. We purified the double-stranded cDNA using AMPure XP beads and subjected it to end repair, addition of the poly-A tail, ligation of the sequencing linker, and fragment size selection. Finally, the cDNA libraries were subjected to PCR enrichment and sequenced with the Illumina HiSeq 2500.

#### PacBio Iso-Seq Library Preparation

For SMRTbell libraries, were combined equal amounts of total RNA from the biological replicates and generated two RNA pools, the heat stress pool and the control pool. From these pools, oligo (dT) was used to enrich for mRNA containing a poly-A tail, then mRNA was reverse transcribed into cDNA using the SMARTer PCR cDNA Synthesis Kit. We used PCR to amplify the cDNAs. The fragments were then screened for large-scale PCR to obtain sufficient cDNA. The full-length cDNA obtained was subjected to injury repair, end-repair, ligation of SMRT dumbbell-type linkers, and construction of a full-length transcriptome library. We removed the sequence of the unligated linker at both ends of the cDNA and then bound the primer and the binding DNA polymerase to form a complete SMRTbell library.

The libraries were then sequenced using PacBio Sequel II System and SMRT. The raw Iso-Seq data were processed on SMRTlink v6.0 software to obtain subread sequences. A circular consistency sequence (CCS) was obtained from the correction between subreads. The full-length sequences that contained a 5′ primer, 3′ primer, and poly-A tail were clustered using the Iterative Isomer Clustering (ICE) algorithm. Finally, the obtained consensus sequence was calibrated using the short-read sequences to obtain a high-quality sequence for subsequent analysis.

### Identification and Functional Annotation of LncRNAs

To investigate the function of genes, we used BLAST (version 2.2.26) (Altschul et al., 1997), HMMER 3.1 (Eddy, 1998), and KOBAS 3.0 (Xie et al., 2011) to search the NR (Li et al., 2002), Nt (Li et al., 2002), KOG (Koonin et al., 2004), COG (Tatusov et al., 2003), Swiss-prot (Amos and Rolf, 2000), KEGG (Minoru et al., 2004), GO (Ashburner et al., 2000), and protein family (Pfam) (Finn et al., 2016) databases. CPC v0.9 (Kong et al., 2007), CNCI v2 (Sun et al., 2013), PLEK v1.2 (Aimin et al. (2014), and Pfam-scan (Finn et al., 2016) were used to identify lncRNAs from the Iso-Seq data (Chao et al., 2018). We use CNCI with default parameters. CPC (set the e-value “1e-10”) was used to assess the extent and quality of the ORF in a transcript and search the sequences with NCBI eukaryotes’ protein database to clarify the coding and non-coding transcripts. Pfam searches use default parameters of −E 0.001 −domE 0.001. The PLEK used to assess coding potential for species that lack high-quality genomic sequences and annotations. PLEK used default parameters of -minlength 200, and removed transcripts <200 bp. Transcripts predicted with coding potential by either/all of the three tools above were filtered out, and those without coding potential were our candidate set of lncRNAs. Since the whole genome sequencing of *P. canadensis* has not yet been completed, lncRNAs were classified into four categories by aligning the mRNA sequences of *P. canadensis* which created by SMRT technology combined with high-throughput sequencing and also considered the *Populus trichocarpa* genome^1^. The lncRNAs that have generic exonic overlap with a known transcript was called sense lncRNAs. Contained in the intergenic lncRNA was called long intervening/intergenic non-coding RNAs (lincRNAs). The lncRNAs that have exonic overlap with known transcripts, but on the opposite strand was called antisense lncRNAs, and fall entirely within an intron of a known gene was called sense intronic lncRNAs (Mercer et al., 2009; Ponting et al., 2009; Roberts et al., 2011; Shuai et al., 2014; Wright, 2014; Chen et al., 2015, 2016). Prediction of the target genes of the lncRNAs were *cis* and *trans* prediction. To predict the target genes of the lncRNAs, we used BLAST (version 2.9.0+) to search our transcriptome libraries, and select a target sequence that was complementary to the lncRNA, setting *E*-value = 1e-5 and identity = 80%. Then, the *cis*-target gene were obtained based on the location relationship between lncRNAs and target genes, and potential target genes (correlation >0.8 or correlation <−0.8, *p* < 0.05) were selected based on the correlation coefficients of lncRNA and mRNA expression where the target gene was called *trans*-target gene (Chen et al., 2015; Li et al., 2017; Mao et al., 2017; Lin et al., 2018).

### Data Analysis

We used RSEM (Dewey and Bo, 2011) to determine transcript levels from the short-read data. The Illumina data was mapped to PacBio data using LoRDEC software to obtain clean reads. To identify gene expression levels in response to heat stress, the redundant sequences were removed using CD-HIT, and the resulting full-length transcripts were used as a reference sequence (ref) for each gene, and then the clean reads of each sample obtained by short-read were aligned to the ref sequence. Next, we obtained the readcount of each gene by mapping the short-read data to the ref. Further, the readcounts were converted into fragments per kilobase of exon model per million mapped reads (FPKM) values.

Differentially expressed genes (DEGs) were selected using log_2_ FC≥ 1 or ≤−1 and FDR <0.05 (*q*-value). Genes with FPKM >0.3 in samples from the two groups were selected for further analysis (Anders and Huber, 2010). All DEGs were mapped to individual terms in the gene ontology (GO) database^2^ and the number of genes per term was calculated. We then used GOseq software for GO enrichment analysis to identify significant enrichment terms in the DEGs. Analysis of gene regulatory pathways was conducted using the KEGG Pathway database^3^.

## Results

### High Quality Full-Length Sequences Were Sufficient for Further Analysis

To reveal the genes and regulatory lncRNAs that respond to heat in *P. canadensis*, we sequenced and analyzed the transcriptomes of the two RNA pools using the PacBio Sequel platform to obtain accurate long reads. A total of 140,046 polymerase reads and 4,297,179 subreads were produced with SMRT, with an average read length of 2,057 bp (Table 1). To provide more accurate sequence information, we obtained 121,801 circular consensus sequences (CCS) from reads that made at least two full passes through the insert (average read length of 2,490 bp) (Table 1).

**TABLE 1:** Summary of reads from third-generation long-read sequencing.

**Item**	**Number**	**Mean length**
Polymerase reads	140,046	65,414
Circular consensus sequences (CCS)	121,801	2,490
Full-length non-chimeric reads (FLNC)	111,807	2,314
Polished consensus	66,657	2,339
The correct polished consensus	66,657	2,336

SMRTlink found 114,373 full-length sequences and 111,807 full-length non-chimeric (FLNC) reads (average read length of 2,314 bp) (Table 1). The similar FLNC reads were clustered using the ICE algorithm. The consensus sequence was obtained, and the non-full-length sequence was corrected by arrow software to obtain the consensus sequence, and finally 66,657 polished consensus sequences were obtained (Table 1), with mean length of 2,339 bp.

Then, the cDNA libraries were sequenced following the Illumina HiSeq 2500 platform paired-end protocol. The RNA-seq generated 187,706,638 and 202,259,974 raw data from the pcq and pch samples, respectively, with 183,393,014 (pcq) and 198,641,402 (pch) cleaned reads remaining after trimming (Table 2). The raw reads of Illumina sequencing data were used to correct the SMRT data. After removing redundant and similar sequences, we obtained 86,014,859 nucleotides and 66,657 transcripts with mean length of 2,336 bp. Transcript length distribution results showed that ^~^1.72, 8.87, 23.6, 42.83, and 22.98% of the transcripts from the corrected isoforms were <500 bp, 500–1,000 bp, 1,000–2,000 bp, 2,000–3,000 bp, and >3,000 bp, respectively. A total of 66,657 corrected consensus sequences were de-redundified using CD-HIT, and 33,840 full-length transcripts were obtained (Table 1).

**TABLE 2:** Summary of Illumina sequencing.

**Samples**		**Raw reads**	**Clean reads**	**Clean bases (G)**
pcq	Average	62,568,879.33	61,131,004.67	9.17
	Total	187,706,638	183,393,014	27.51
pch	Average	67,419,991.33	66,213,800.67	9.93
	Total	202,259,974	198,641,402	29.79

### High-Quality Full-Length Sequences Improved the Accuracy of LncRNA Prediction

After the corrected consensus sequences were de-redundified using CD-HIT, the 33,840 full-length transcripts were used as reference sequences for the genes. Then the clean reads of each sample obtained by Illumina sequencing were aligned to the ref, and the readcounts of genes were obtained based on the mapping results. The number of mapped reads in the six libraries ranged from 46,427,856 to 61,084,944, and the mapping rates ranged from 86.52 to 89.44% (Supplementary Table S1). In this process, we used RSEM software, and set the parameters of the comparison software bowtie2 in RSEM to end-to-end and sensitive modes, and the others were the default parameters.

**FIGURE 1:**
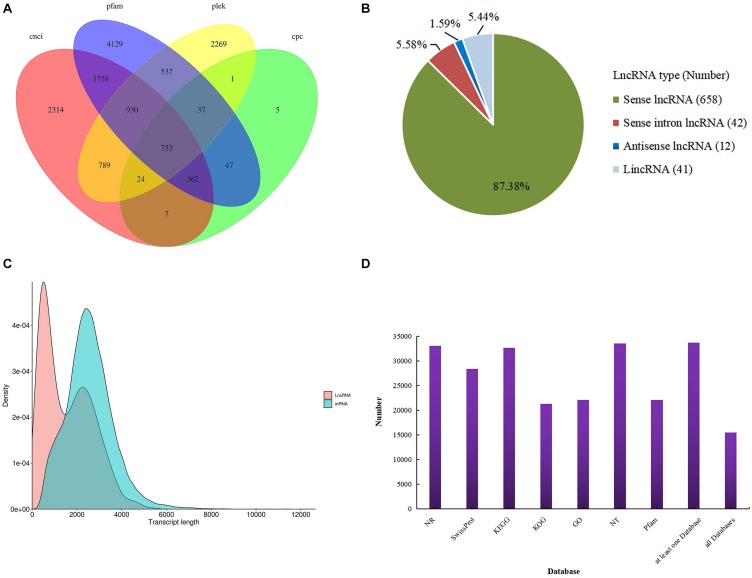
Identification of lncRNAs. **(A)** Venn diagram of lncRNAs predicted by CNCI, CPC, Pfam, and PLEK methods. **(B)** Proportions of four types of lncRNA. **(C)** Density and length distributions of mRNAs and lncRNAs in *P. canadensis*. **(D)** Seven database annotation result statistics.

For the identification of lncRNAs, we combined the two transcript datasets and selected for transcripts longer than 200 bp and lacking potential coding capability; this predicted lncRNAs was 753 (Figure 1A). We classified these lncRNAs into four groups: 658 sense lncRNAs (87.38%), 41 long intervening/intergenic non-coding RNAs (lincRNAs) (5.44%), 12 antisense lncRNAs (1.59%) and 42 sense intronic lncRNAs (5.58%) (Figure 1B). Analysis of mRNA and lncRNA sequence lengths showed that the average length was 2,564 and 1,568 bp. (Figure 1C). In addition, all 33,840 transcripts (corrected isoforms) were functionally annotated by searching NR, Swissprot, GO, COG, KOG, Pfam, and KEGG databases and a total of 33,676 transcripts (99.5%) were annotated (Figure 1D). We identified the largest number of homologs in *P. trichocarpa* by comparing the transcript sequences of *P. canadensis* to the NR database (Supplementary Table S2). Lastly, the Illumina sequencing data were remapped with the SMRT data. The resulting full-length transcripts were used as a ref, and all valid reads were obtained from mapping short-read sequences to ref using RSEM software. After mapping, we converted the readcounts into FPKM to measure the transcript level for each unigene.

### Analysis of Differentially Expressed Genes and LncRNAs

**FIGURE 2:**
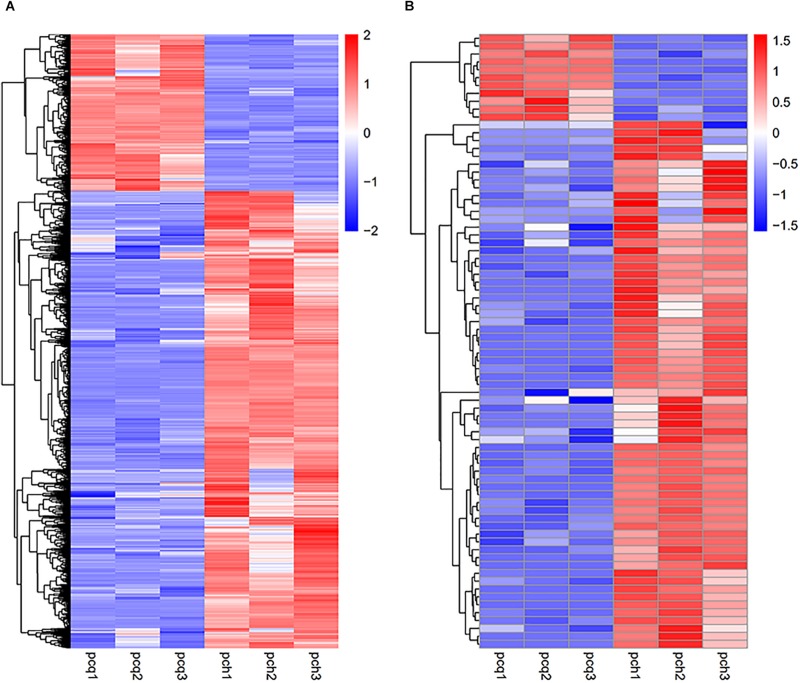
Expression profiles of genes and lncRNAs. Hierarchical clustering of all DEGs **(A)** and lncRNAs **(B)** in the *P. canadensis* pcq and pch groups.

**FIGURE 3:**
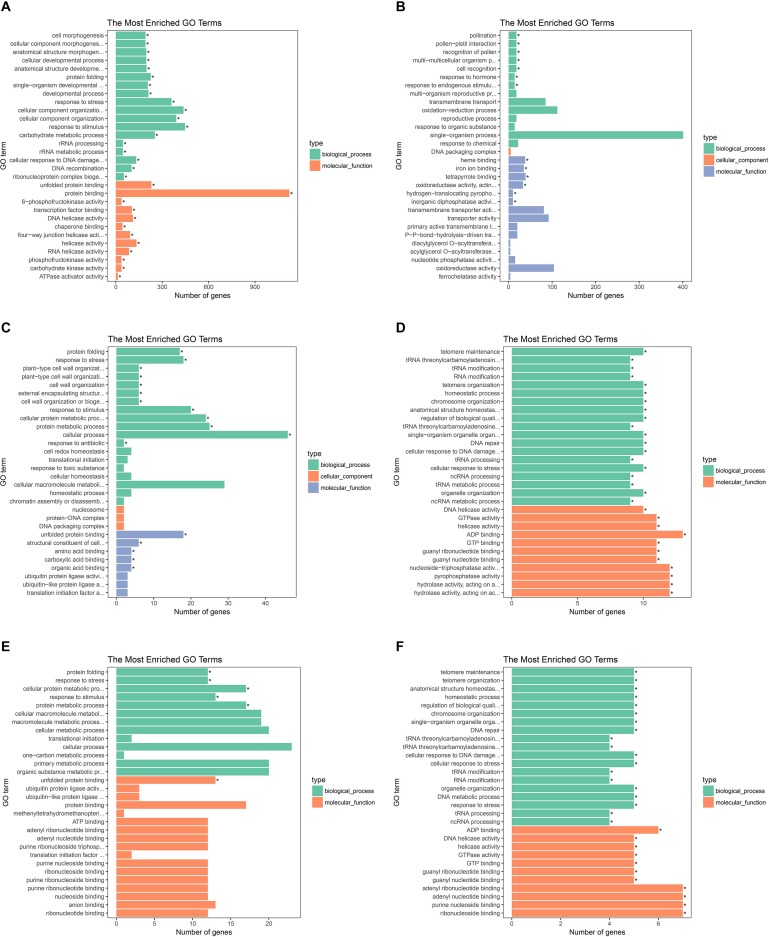
GO analysis of the biological function of genes, lncRNAs, and target genes of lncRNAs. GO terms of up- **(A)** and down-regulated genes **(B)**, up- **(C)**, and down-regulated lncRNAs **(D)**. And up- **(E)** and down-regulated target genes of lncRNAs **(F)**.

**FIGURE 4:**
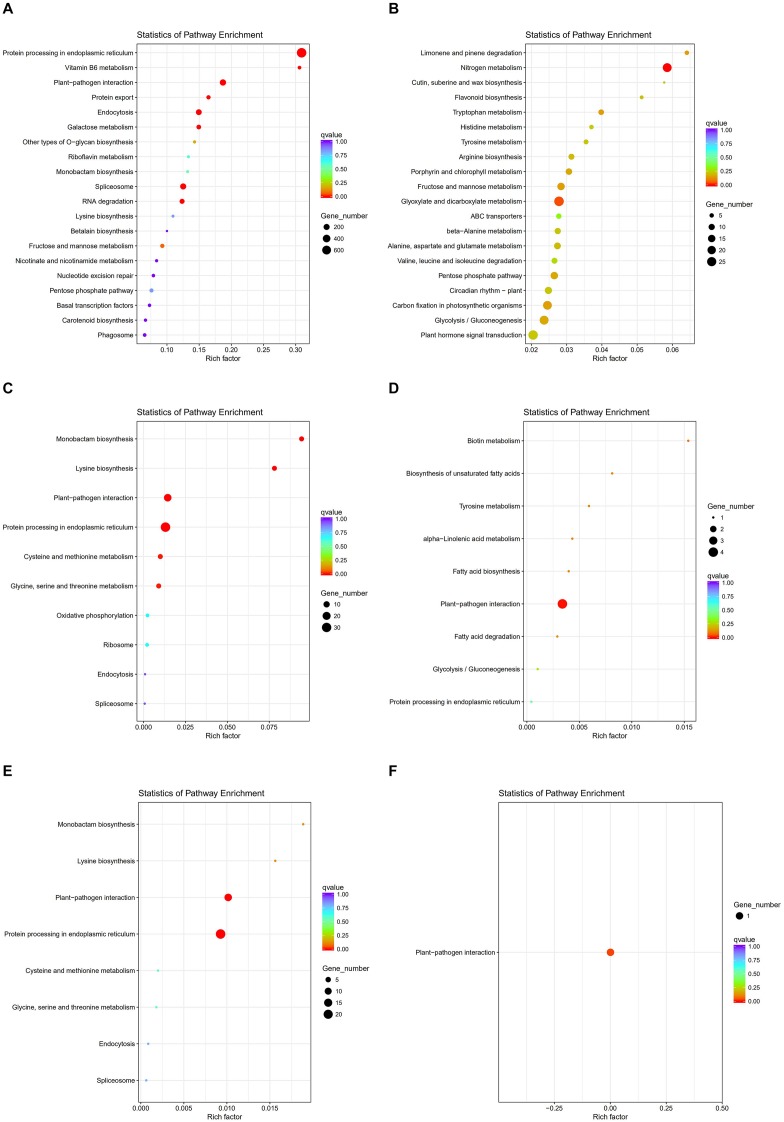
KEGG pathway enrichment analysis of genes, lncRNAs, and target genes of lncRNAs. KEGG pathway of up- **(A)** and down-regulated genes **(B)**, up- **(C)** and down-regulated lncRNAs **(D)**. and up- **(E)** and down-regulated target genes of lncRNAs **(F)**.

To investigate gene expression patterns in *P. canadensis*, we used FPKM values to measure the transcript level of every unigene and compared the control and the heat-stress-treated groups. This analysis detected 3,493 genes as differentially expressed based on SRMT sequencing (Figure 2A). Among them, 2,601 and 892 were up-regulated and down-regulated, respectively, in the pch group compared with the pcq group. GO enrichment analysis of significantly DEGs showed that the up-regulated genes were associated with 171 biological processes such as response to stimulus, 70 molecular functions such as unfolded protein binding and heat shock protein binding, and 51 cellular components such as transmembrane transporter complex (Figure 3A). Moreover, down-regulated genes were associated with seven biological processes such as response to endogenous stimulus and response to hormones, and six molecular functions such as heme binding, but the cellular components were not found to be significantly enriched (Figure 3B). KEGG pathway enrichment analysis applied on the differentially expressed mRNAs showed that the up-regulated genes were associated with 10 pathways such as protein processing in the endoplasmic reticulum (Figure 4A) and down-regulated genes were associated with 18 pathways such as plant hormone signal transduction (Figure 4B).

Moreover 78 significantly differentially expressed lncRNAs were obtained, including 67 up-regulated lncRNAs and 11 down-regulated lncRNAs (Figure 2B). We identified 923 candidate target genes of lncRNAs, and 72 significantly differentially expressed target genes. GO enrichment analysis of target genes of lncRNAs showed that the up-regulated target genes were associated with 12 biological processes such as protein folding and response to stress, and five molecular functions such as unfolded protein binding and heat shock protein binding, but the cellular components were not found to be significantly enriched (Figure 3C). Moreover, down-regulated target genes were associated with 45 biological processes such as cellular response to stress, 33 molecular functions such as ADP binding, and 26 cellular components such as cytoskeletal part (Figure 3D). KEGG pathway enrichment analysis applied to the target genes showed that the up-regulated target genes were associated with six pathways such as protein processing in the endoplasmic reticulum (Figure 4C) and down-regulated target genes were associated with four pathways such as biotin metabolism (Figure 4D).

### LncRNAs Regulate Genes in Response to Heat Stress in *P. canadensis*

**FIGURE 5:**
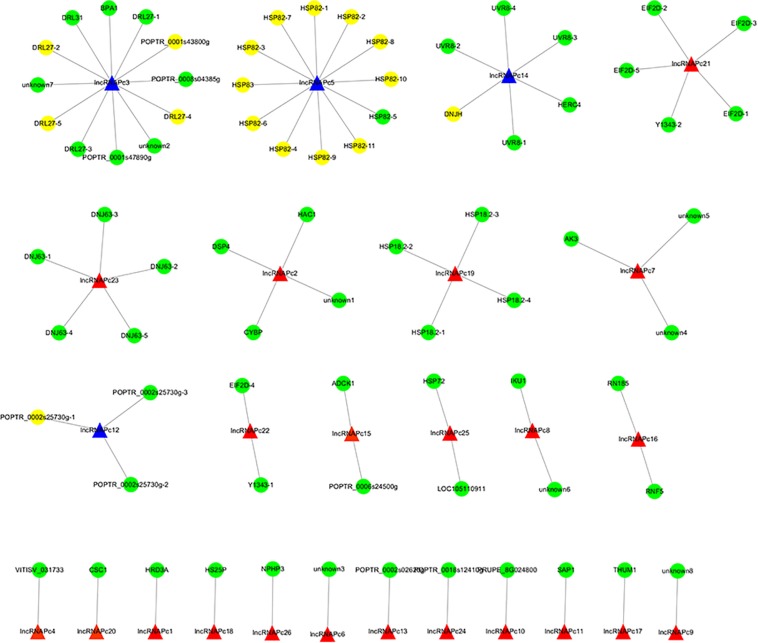
The lncRNA–gene interaction networks. The blue triangle is the candidate lncRNA and the yellow circle is the genes.

The correlation between lncRNAs and gene expression was calculated by Pearson correlation coefficient, and the interaction network between lncRNAs and target genes was constructed (Figure 5). GO enrichment analysis showed that the up-regulated target genes were associated with five biological processes such as protein folding and response to stress, and one molecular function (unfolded protein binding), but the cellular components were not found to be significantly enriched (Figure 3E). Moreover, down-regulated genes were associated with 33 biological processes such as cellular response to stress, 31 molecular functions such as ADP binding, and 17 cellular components such as mitotic spindle (Figure 3F). KEGG pathway enrichment analysis applied on the differentially expressed target genes showed that the up-regulated genes were associated with three pathways such as protein processing in the endoplasmic reticulum (Figure 4E) and down-regulated genes were associated with one pathway, which was plant-pathogen interaction (Figure 4F). Furthermore, 19 genes in the heat stress-related GO term were annotated, including *HSP82*, *HSP83*, *DRL27*, *DNJH*, and two predicted protein-coding genes (POPTR_0001s43800g and POPTR_0002s25730g), and these genes are the target gene of four sense lncRNAs (lncRNAPc3, lncRNAPc5, lncRNAPc12, and lncRNAPc14) (Table 3). In addition, four lncRNAs were aligned to the *P. trichocarpa* transcriptome data by sequence alignment, and the results showed that the genes with the highest ratio of alignment were *DRL*, *UVR8*, *NDUA9*, and *HSP83*.

**TABLE 3:** The DEGs in GO term related to heat stress.

**Gene name**	**Readcount (heat treatment)**	**Readcount (control)**	**log_2_FC**	**pval**	**padj**
*DNJH*	1,740.172	6.651066	8.0314	3.33 × 10^–16^	1.54 × 10^–13^
*DRL27-2*	50.92479	634.6025	−3.6394	9.44 × 10^–5^	0.00264
*DRL27-4*	897.1711	6,233.571	−2.7966	0.00159	0.0267
*DRL27-5*	1,694.13	18,919.97	−3.4813	0.00145	0.0250
*HSP82-1*	2,057.985	10.19488	7.6572	4.06 × 10^–15^	1.48 × 10^–12^
*HSP82-2*	5,836.55	5.336274	10.095	3.64 × 10^–17^	2.16 × 10^–14^
*HSP82-3*	402.9229	3.697016	6.768	2.08 × 10^–13^	5.37 × 10^–11^
*HSP82-4*	3,894.566	44.37201	6.4557	6.43 × 10^–11^	9.45 × 10^–9^
*HSP82-6*	169.0849	1.005348	7.3939	1.26 × 10^–7^	8.56 × 10^–6^
*HSP82-7*	714.3319	0	Inf	5.38 × 10^–15^	1.92 × 10^–12^
*HSP82-8*	39.02773	0	Inf	2.49 × 10^–7^	1.55 × 10^–5^
*HSP82-9*	249.306	0	Inf	1.40 × 10^–16^	7.00 × 10^–14^
*HSP82-10*	685.5355	0	Inf	3.44 × 10^–11^	5.43 × 10^–9^
*HSP82-11*	17,017.61	51.03778	8.3812	3.03 × 10^–9^	3.06 × 10^–7^
*HSP83*	150.3455	0.758881	7.6302	4.29 × 10^–11^	6.56 × 10^–9^
POPTR_0001s43800g	4.550953	136.762	−4.9094	9.09 × 10^–7^	4.87 × 10^–7^
POPTR_0002s25730g	448.0703	4.377071	6.6776	0.00218	0.0343

## Discussion

### Full-Length Sequences Identified by SMRT Sequencing in *P. canadensis* Improved Studies of the Heat Stress Response

The emergence of SMRT sequencing has greatly facilitated the *de novo* assembly of transcriptomes in higher organisms (Au et al., 2013; Sharon et al., 2013). In *Populus tomentosa*, short-read sequences obtained from Illumina sequencing showed that the average length of transcripts was 690, 703, and 686 bp in normal, tension, and opposite wood, respectively (Chen et al., 2015), and the average length of transcripts was 671 and 356 bp in *Populus euphratica* and *P. trichocarpa* × *Populus deltoides*, respectively (Qiu et al., 2011; Benjamin et al., 2012). In our study, we first used high-throughput sequencing to obtain high-quality short sequence reads of the *P. canadensis* transcriptome. Then, we used SMRT sequencing to obtain full-length sequence reads with an average length of 2,339 bp. The sequences obtained by third-generation sequencing were longer than those obtained by second-generation sequencing. In part of lncRNA identification, most of the identified lncRNAs are <2,000 bp in length in our study. The length of lncRNAs were less than the average length obtained by third-generation sequencing, indicating that our method can obtain more accurate lncRNA sequences compared to RNA-seq. This is consistent with the research reported by Wang B. et al. (2016). Furthermore, we explored transcriptomic changes in *P. canadensis* in response to heat stress by combined SMRT sequencing with Illumina sequencing and demonstrated that this approach provides higher quality reads and more complete assembly compared with using short reads alone (Au et al., 2013; Sharon et al., 2013; Huddleston et al., 2014). We obtained 66,657 complete transcript sequences for *P. canadensis* by using short reads to correct long reads of SMRT technology sequencing, and greatly improved the accuracy and depth of the study. This is the first study using PacBio Iso-Seq to examine the full-length transcriptome of *P. canadensis*. The obtained transcriptome may help to further explore *P. canadensi*s genetics and the association of gene expression with phenotypes.

### Gene Expression of *P. canadensis* in Response to Heat Stress

Plants have complex regulatory networks that help them to acclimatize to heat stress (Reddy et al., 2016). Analysis of heat-stress-responsive genes can improve our understanding of the response to heat stress. In this study, we identified 3,493 DEGs that reflect changes in gene expression in *P. canadensis* heat stress responses and these DEGs provide a foundation for further research. Our GO and KEGG enrichment analysis of these DEGs identified biological processes, metabolic pathways, and biochemical activities that are up- or down-regulated in response to heat, thus providing an in-depth analysis of the transcriptome changes that occur during the heat-stress response of *P. canadensis*. DEGs associated with high temperature stress in *P. canadensis* were enriched in the GO terms single-organism metabolic process, oxidation-reduction process, and transmembrane transport biological process. Further study of these DEGs will help us to elucidate the mechanisms involved in heat tolerance and to identify new, potentially useful heat-stress-related genes specific to *P. canadensis*.

These results were supported by the reports of previous studies (Liu, 2014). Furthermore, we annotated genes that were significantly associated with heat stress with GO terms, and the results showed that many transcription factor-related genes, FtsH protease family genes, HSP family genes, and endopeptidase Clp family genes (including *TFB2*, *FTSH6*, *HSP90*, *HSP83*, *HSP82*, and *CLPB1*) were enriched. Among them, *HSP90*, *HSP83*, and *HSP82* have been reported to participate in the heat stress response in previous studies (Borkovich et al., 1989; Gkouvitsas et al., 2009; Zhang et al., 2013).

### LncRNAs Regulate Genes in Response to Heat Stress in *P. canadensis*

There are many molecular mechanisms regulating gene expression, including *cis*-regulatory elements and *trans*-acting factors such as lncRNAs (Lasky et al., 2014; Albert and Kruglyak, 2015). Our study identified 753 putative lncRNAs in the pch and pcq samples by full-length transcriptome sequencing and classified them into four classes to explore their possible functions. Since the whole genome of *P. canadensis* has not been sequenced, the genome of *P. trichocarpa* was used as a reference in our study. The lncRNAs we identified were shorter than the mRNAs, in agreement with previous studies (Pauli et al., 2012). LncRNAs can be significantly differentially expressed in plants under heat stress, for example, Di et al. (2014) found that 245 poly (A)^+^ and 58 poly(A)^–^ lncRNA have shorter lengths and lower expression levels than mRNAs under heat stress. In our study, 78 lncRNAs showed significant differential expression after heat stress, suggesting that lncRNAs may be involved in the plant high temperature stress responses. However, the regulatory role of these lncRNAs is unclear, and further analysis will be needed to explore this.

LncRNAs can regulate gene expression (Liu et al., 2012). In our study, 923 target genes were obtained by *trans* prediction. The correlation between lncRNA and gene expression was calculated by Pearson correlation coefficient, and the interaction network between lncRNAs and target genes was constructed. The results of GO enrichment and KEGG pathway analysis indicated that the up-regulated lncRNA target genes were significantly associated with heat stress, suggesting that lncRNAs may regulate target genes in response to heat stress. Further, 19 genes in the heat stress-related GO term were annotated, including *HSP82*, *HSP83*, *DRL27*, *DNJH*, and two predicted protein-coding genes (POPTR_0001s43800g and POPTR_0002s25730g), and these genes are the target genes of four sense lncRNAs. Among them, *HSP82* and *HSP83* are the target genes of the sense lncRNAPc5. Further, lncRNAPc5 aligned to *P. trichocarpa HSP83*, which shows that these lncRNAs identified from full-length transcriptome sequencing may regulate HSP genes by co-expression. These results suggested that lncRNAPc5 may *cis*-regulate HSP genes via co-expression. Exploration of the functions of the identified lncRNAs will provide further insight into the heat-stress response of *P. canadensis*.

## Data Availability Statement

The raw sequence data reported in this article have been deposited in the Genome Sequence Archive (Genomics, Proteomics and Bioinformatics, 2017) in BIG Data Center (Nucleic Acids Research, 2019), Beijing Institute of Genomics (BIG), Chinese Academy of Sciences, under accession numbers CRA002150 and CRA002154 that are publicly accessible at https://bigd.big.ac.cn/gsa.

## Author Contributions

JC designed the research. JX, MF, ZL, MZ, XL, and YP performed the research. JC analyzed and interpreted the data. JC and JX wrote the paper. YW provided critical revision of the paper. All authors commented on the manuscript.

## Conflict of Interest

The authors declare that the research was conducted in the absence of any commercial or financial relationships that could be construed as a potential conflict of interest.

## Footnotes

1 http://www.phytozome.net/poplar

2 http://www.geneontology.org/

3 http://www.genome.jp/kegg/pathway.html
