# Supplementary figures and images for: Third-Generation Sequencing Reveals LncRNA-Regulated HSP Genes in the Populus x canadensis Moench Heat Stress Response
Source: Front Genet. 2020 May 7;11:249. doi: 10.3389/fgene.2020.00249 (PMC7221187; doi:10.3389/fgene.2020.00249)

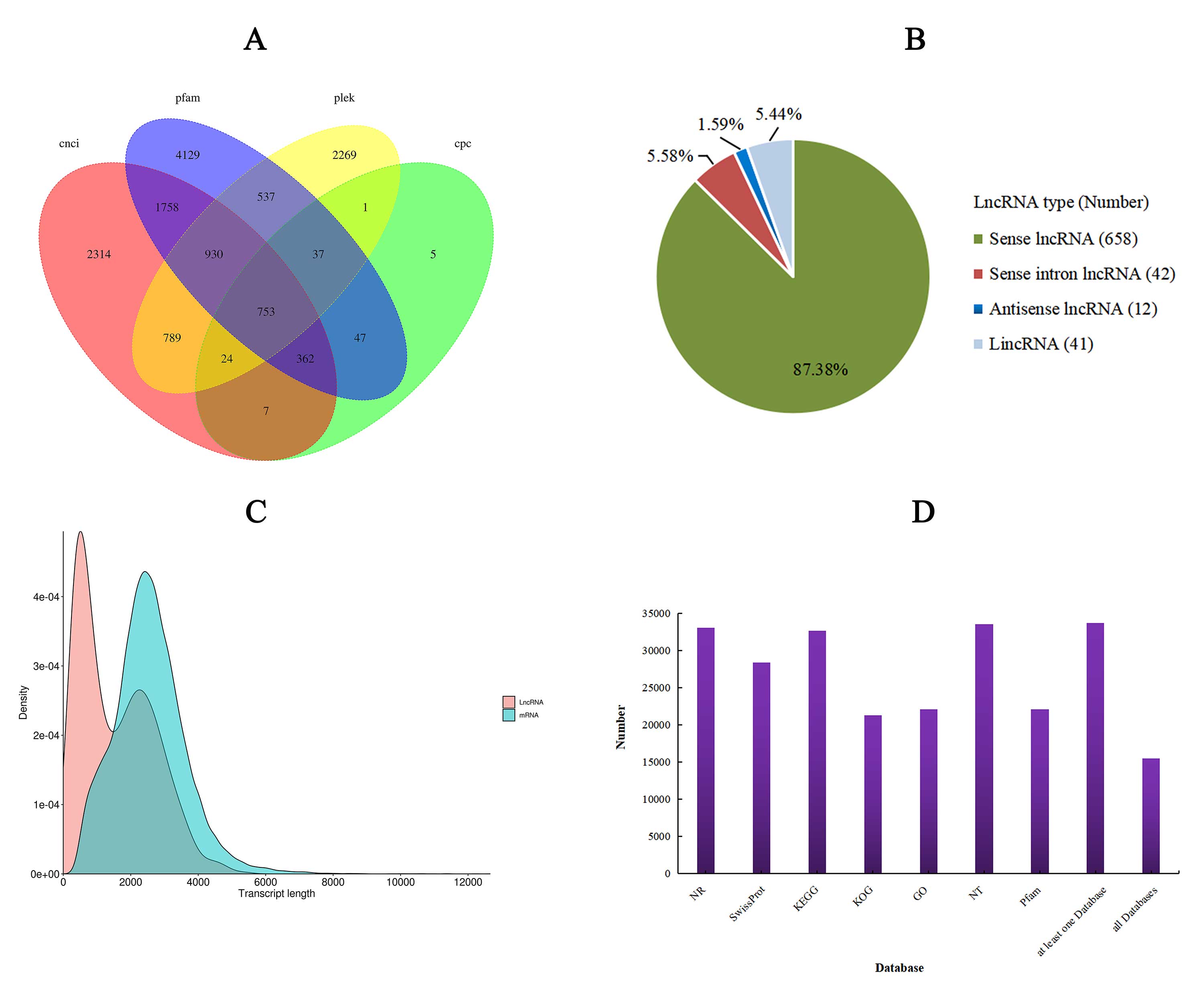

Supplement: Supplementary file 1 [file Figure_1.jpg]

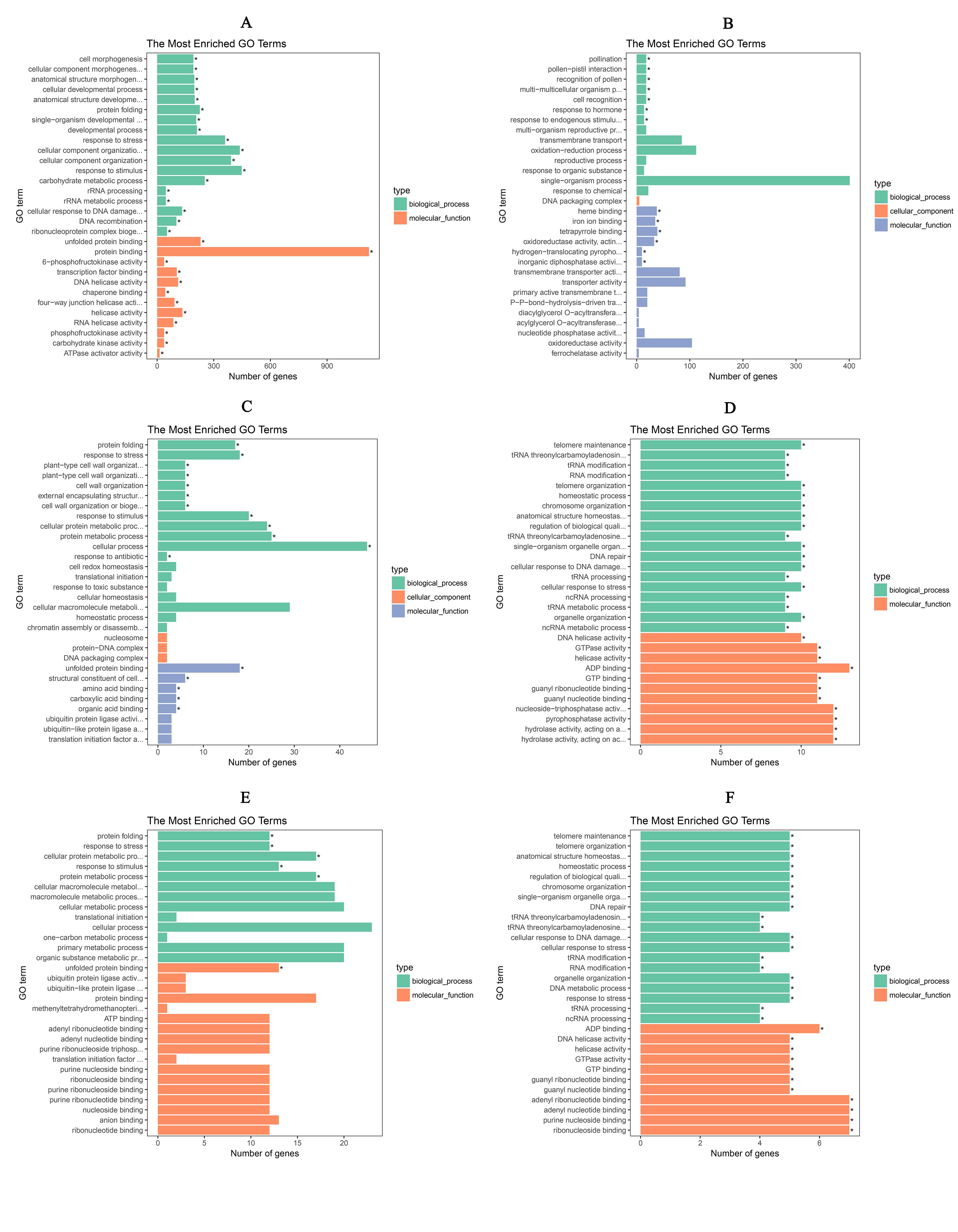

Supplement: Supplementary file 2 [file Figure_3.jpg]

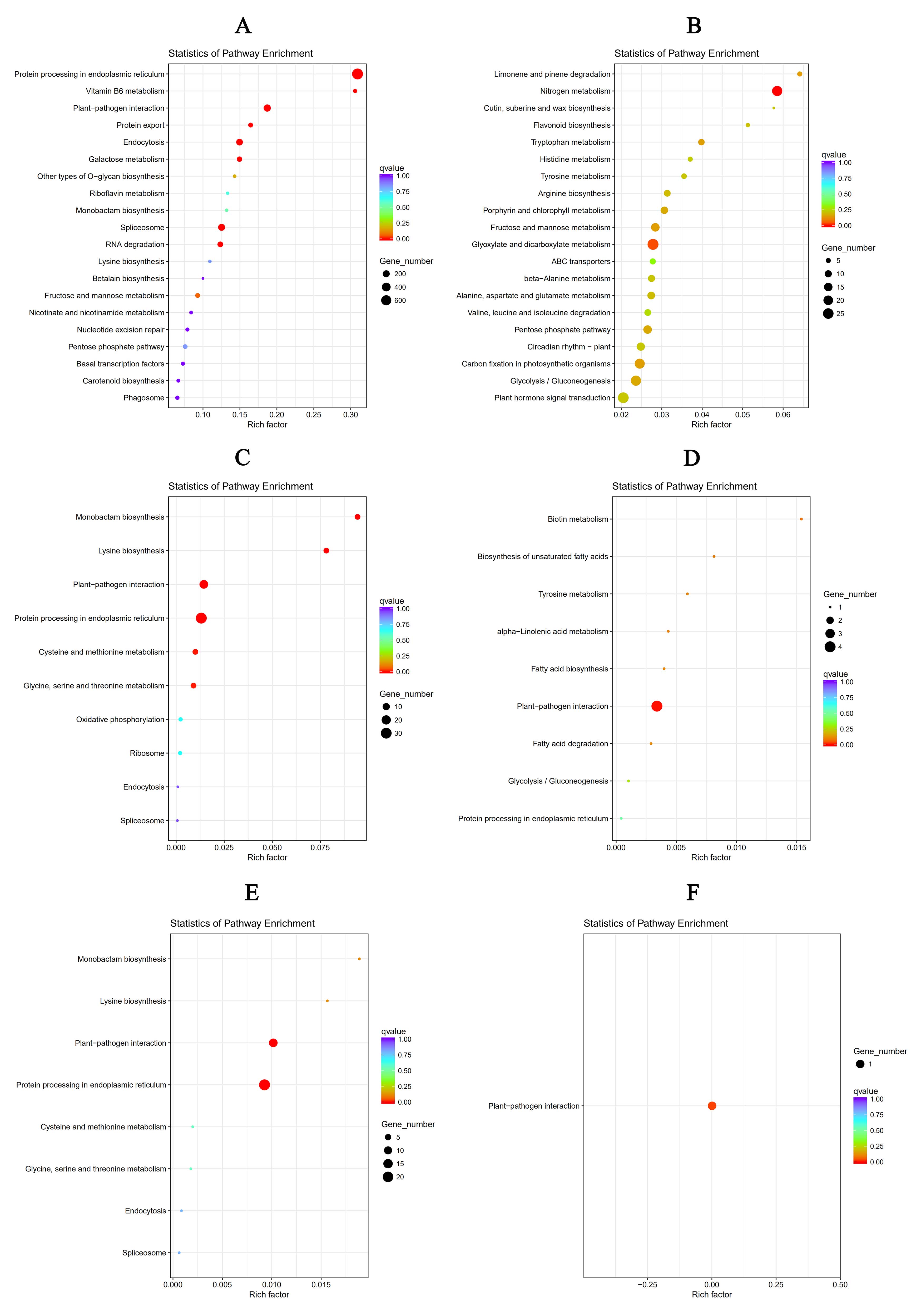

Supplement: Supplementary file 3 [file Figure_4.jpg]
